# Cataloging variation in 16S rRNA gene sequences of female urobiome bacteria

**DOI:** 10.3389/fruro.2023.1270509

**Published:** 2024-01-08

**Authors:** Genevieve Baddoo, Adriana Ene, Zubia Merchant, Swarnali Banerjee, Alan J. Wolfe, Catherine Putonti

**Affiliations:** ^1^ Bioinformatics Program, Loyola University Chicago, Chicago, IL, United States; ^2^ Department of Biology, Loyola University Chicago, Chicago, IL, United States; ^3^ Department of Mathematics and Statistics, Loyola University Chicago, Chicago, IL, United States; ^4^ Department of Microbiology and Immunology, Stritch School of Medicine, Loyola University Chicago, Maywood, IL, United States

**Keywords:** urobiome, 16S rRNA gene sequence analysis, female urinary tract, urinary microbiome, 16S rRNA gene sequence

## Abstract

Continued efforts to isolate and sequence bacteria of the urinary tract has increased representation of these species in publicly available databases. This in turn has improved taxonomic classifications of the urinary microbiome (urobiome). Short-read sequencing targeting a variable region(s) of the 16S rRNA gene sequence has been fundamental in characterizing the urobiomes of males and females with and without lower urinary tract symptoms, as well as cancers of the urinary tract. Here, we have compiled a data set of full-length or near-full-length 16S rRNA gene sequences for the urobiome. To generate this data set, we first plated 203 isolates from the bladder on differential media and sequenced their full-length 16S rRNA gene sequence. We combined this data set with publicly available genomes from primarily the female urinary tract. The final data set includes 399 sequences representative of 160 different species from 73 genera. We assessed the ability of publicly available databases to correctly predict these sequences based on the V1-V3, V4, and V4-V6 variable regions. As expected, species designations based upon these variable regions is often not possible or incorrect. We also detected incorrect genus-level classifications. This data set can be used to supplement existing databases, by increasing urobiome species variation, and thus improve future studies characterizing urobiomes.

## Introduction

High-throughput sequencing technologies were pivotal in first identifying the presence of bacterial DNA in the healthy urinary tract of both males and females (1–4). Improved culture-based techniques, such as the Expanded Quantitative Urinary Culture (EQUC) method (5), enabled researchers to isolate and subsequently sequence more species inhabiting the urinary tract. In the decade that has followed the discovery of the urinary microbiome (urobiome), numerous studies investigating the bacterial fraction of this community have been conducted (see reviews (6, 7)). Whole genome sequencing of strains isolated from the urinary tract have improved representation of urinary taxa in publicly available databases, which has in turn improved bioinformatic assignment of taxa in urobiome studies.

Most urobiome studies have relied on high-throughput sequencing of variable regions of the 16S rRNA gene sequence. Previously targeted variable regions for the urobiome include V1-V2 (8, 9), V1-V3 (1, 3, 10–12), V3-V4 (13–16), V4-V6 (17, 18), V4 (19–30), and V6 (2, 31). General guidelines for 16S surveys of urobiome samples have been recommended (32). Hoffman and colleagues assessed the GreenGenes, SILVA, and NCBI 16S Microbial databases for their ability to assign taxa to the 16S variable regions extracted from the whole genomes of 149 bladder strains, representing 78 species (33). The authors found that the V2-V3 and V1-V3 regions from this *in silico* experiment, when combined with the NCBI 16S Microbial database, correctly identified most bladder bacterial species. The V4 region, which is widely used, also fared well, correctly classifying two-thirds of the bacterial species. This performance was impacted by limitations in the representation of 16S rRNA gene sequence diversity among urinary microbes in the database; furthermore, prior studies have shown that different species of common urobiome genera can have full-length 16S rRNA gene sequences that are nearly identical (34–36). Additionally, when current bioinformatic tools and databases are applied to older studies, different taxonomic assignments have been identified, highlighting the importance of adding urinary-derived isolates to reference databases (37).

Here, we have focused on developing a reference set of full-length or near-full-length 16S rRNA gene sequences for the urobiome. With this reference set, we assess the resolution possible when the frequently used V1-V3, V4, and V4-V6 regions are targeted. The selection of these three regions was informed by prior assessment of the taxonomic resolution possible for bacterial species of the female urinary tract (33). Based upon our analysis, we identify taxa for which species resolution is possible, taxa for which only genus resolution is possible, and taxa for which the genus cannot be identified. In particular, we focus our discussion on taxa associated with symptoms and “health.” Thus, urobiome researchers can integrate prior studies with limitations in mind.

## Methods

### Strain sequencing

Bacterial strains were isolated from urine samples collected via transurethral catheterization as part of prior IRB-approved studies (Loyola: 206439, 209545, 206449; University of California, San Diego: 170077AW) that are described in detail elsewhere (25, 38–41). Briefly, the expanded quantitative urine culture (EQUC) process was performed in the Wolfe lab, as described previously (5). Unique colony morphologies were replated and identified via MALDI-TOF prior to storage at -80°C.

For each isolate examined here, the sample was streaked onto either an anaerobe 5% sheep blood (ANA) agar plate, 5% sheep blood agar plate (BAP), or nalidixic acid (CNA) agar plate, depending upon the genus identified by MALDI-TOF. (All three plates were BD BBL prepared plated media [Becton, Dickinson and Co., Sparks, MD].) Each plate was incubated for 48 h in 5% CO_2_ at 35°C. All colonies were scraped off of the surface of the plate and added to 1 mL of liquid media. The media was selected based upon the genus predicted by MALDI-TOF; these media include: Lysogeny broth (LB), Actinomyces broth (Sigma-Aldrich), Brain Heart Infusion broth (BHI) (BD) + 1% Tween 80 (BHI+Tween), De Man, Rogosa, and Sharpe broth (MRS) (Millipore) + 1% Tween 80 (MRS+Tween), New York City III broth (NYC III), Tryptic Soy broth (TSB) +5% sheep blood, or TSBYE (TSB+0.5% w/v yeast extract). Liquid cultures were incubated for 48 h at 35°C with 5% CO_2_, after which bacterial glycerol stocks were created using 1 mL liquid culture and 1 mL 50% (v/v) glycerol. Stocks were then frozen at -80°C until further processing.

Each freezer stock was streaked onto 6 different types of 1.7% agar plates (LB, Actinomyces, TSB, NYC III, BHI+Tween, MRS+Tween) and incubated for 48 h in 5% CO_2_ at 35°C. Morphologically distinct colonies were identified using a light microscope, and a single colony of each morphology was picked from each plate, added to 1 mL of the liquid media of the plate from which it was derived, and incubated for 48 h in 5% CO_2_ at 37°C.

DNA was extracted from the liquid culture of the colony using the DNeasy UltraClean Microbial Kit (Qiagen, Hilden, Germany), following the manufacturer’s protocol. DNA concentration was quantified using the Qubit fluorometer (ThermoFisher Scientific, Waltham, MA USA). The 16S rRNA gene sequence was amplified using the 63f (5′-CAG GCC TAA CAC ATG CAA GTC-3′) and 1387r (5′-GGG CGG WGT GTA CAA GGC-3′) primers (42). PCR products were purified from the reaction mixture using the E.Z.N.A. Cycle Pure Kit (Omega Bio-tek, Inc., Norcross, GA USA), following the manufacturer’s protocol, and quantified using the Qubit fluorometer. 16S rRNA gene amplicons were sequenced via Sanger sequencing by Genewiz from Azenta Life Sciences (New Brunswick, NJ, USA) with 2x coverage.

Each forward and reverse read was manually trimmed and assembled in Geneious v. 2021.0.3 (Biomatters, Ltd., Auckland, New Zealand). Sequences were filtered out if they contained 30 or more unassigned nucleotides (Ns) and/or gaps in their assembly. The resulting sequence was queried against the NCBI 16S ribosomal RNA sequences database via megablast (as of April 2022). Hits were manually inspected, and taxonomic classification was made. If two or more identical sequences were generated for isolates from the same sample (signifying that the same species was isolated on two or more medias and/or two or more colonies that were perceived to be morphologically distinct), only one was kept for further analysis; the longer of the two was retained. Taxonomic designations were confirmed in December 2023 via the NCBI Taxonomy database. Genera and species listed in the results and supplemental materials reflect the most up to date names.

### Creating a unique set of 16S rRNA sequences from bladder bacteria

First, all rna_genomic files for genome assemblies in the BioProject PRJNA316969 were retrieved (as of April 18, 2022). The genomes within this BioProject are predominately from bladder urine, collected via transurethral catheterization, primarily from adult females; non-bladder isolates, determined by referring to metadata records, were removed from the data set. **Supplementary Table S1** lists metadata for the strains included in this data set, as well as the strains from our own sequencing efforts. Multiple sequence alignment was performed using MAFFT v7.490 (43) through Geneious Prime v2022.1.1 (Biomatters Ltd., Auckland, NZ). Duplicate sequences were removed, as were sequences that were subsequences of a longer representative sequence.

### Variable region simulation

Full-length 16S rRNA gene sequences were used to derive sequences representative of sequencing the V1-V3, V4, and V4-V6 regions. **Table 1** lists the primers used for these regions. The same procedure used by Hoffman et al. (33) was employed here by using their script (https://github.com/lakarstens/BladderBacteriaSpecies/tree/master/split_to_vr). For downstream analyses, the fasta files were converted to fastq files using seqtk seq tools (https://github.com/lh3/seqtk). Fastq files were analyzed using DADA2 (44). The assignTaxonomy function from DADA2 was used to assign taxonomy with the SILVA database (v138.1) (45). Full-length 16S rRNA gene sequences were retrieved from SILVA, which includes relevant updates from the GTDB taxonomy.

**Table 1:** Primer sequences used to simulate short read sequencing of bladder bacteria.

Variable Region	Primer F	Primer R
V1-V3	27F: AGAGTTTGATCCTGGCTCAG	534R: ATTACCGCGGCTGCTGG
V4	515F: GTGCCAGCMGCCGCGGTAA	806R: GGACTACHVGGGTWTCTAAT
V4-V6	515F: GTGCCAGCMGCCGCGGTAA	1114R: GGGGTTGCGCTCGTTGC

### Phylogenetics

Phylogenetic trees were determined as follows. Sequences were imported into Geneious Prime and aligned using the MAFFT (43) plug-in through Geneious Prime. The phylogenetic tree was derived using the FastTree v2.1.12 (46) plug-in with default parameters through Geneious Prime and visualized using iTOL v6 (47).

## Results

Of the 203 bladder isolates plated, 155 had growth on at least one of the six media used here (see Methods). DNA was extracted from 1,008 morphologically distinct single colonies from these plates and 16S rRNA gene sequencing was performed resulting in 831 high-quality sequences (**Supplementary Data Sheet S1**). Taxonomy was assigned via BLAST against NCBI’s 16S ribosomal RNA sequences database. These near-full length sequences range in length from 811 to 1319 bp (average 1250 bp). These 16S rRNA sequences represent 69 different species and 33 genera were identified.

**Figure 1:**
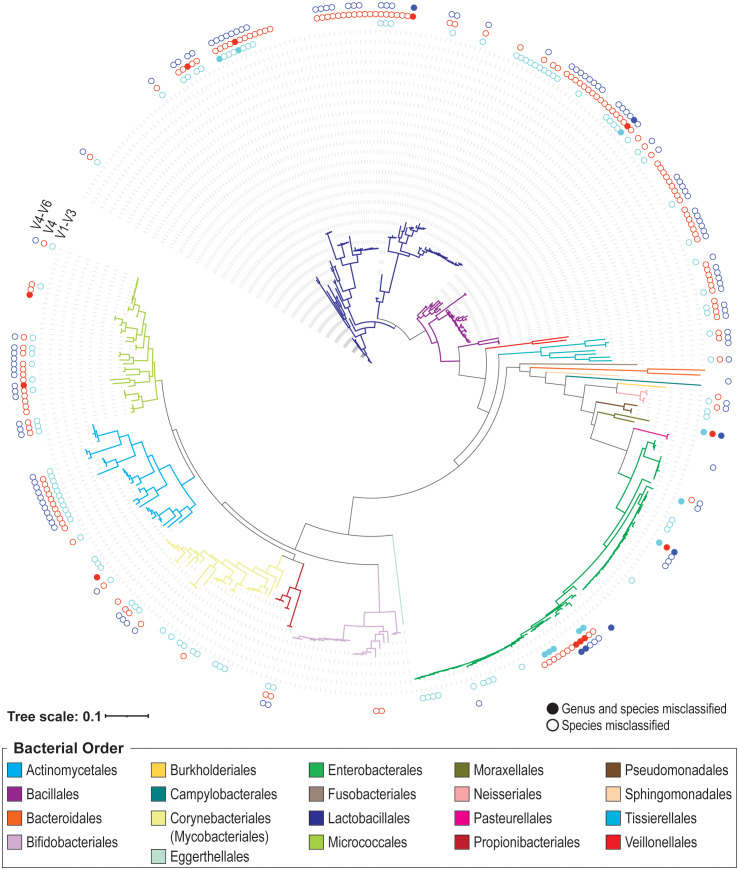
Phylogenetic tree of species included in the 399 urinary 16S rRNA gene sequence data set. Branches are colored according to their bacterial order, as indicated in the legend. The outer rings indicate if the V1-V3 (inner, aqua), V4 (middle, red), and/or V4-V6 (outer, indigo) genus/species predictions are correct. If the variable region identifies the correct genus and species, no dot is drawn. If the genus prediction is correct, but the species is not, the dot is an open circle. If neither the genus nor species prediction is correct, the dot is filled.

We next built a 16S rRNA gene sequence data set representative of the phylogenetic diversity within the bladder. We supplemented the sequences generated from our 16S rRNA gene sequencing of bladder isolates with publicly available urinary strains were added. Most of these strains were isolated from catheterized urine samples from females. In total, this data set includes 399 unique sequences – 98 from the full-length 16S rRNA gene sequencing performed here. (Other full-length 16S rRNA gene sequences from the plated isolates were better represented by sequences from the publicly available sequences.) These 399 sequences are provided in **Supplementary Data Sheet S2** and associated metadata is available in **Supplementary Table S1**. The 399 sequence data set represents 160 different species from 73 genera (**Supplementary Table S2**). While these sequences range in size from 1,139 bp to 1,576 bp, all but five exceed 1,200 bp in length. **Figure 1** presents the phylogenetic diversity of the species included in this data set. Additionally, the 399 sequence data set includes 5 sequences (all from prior sequencing efforts) that are assigned a genus, but the species has yet to be resolved: *Bacillus* sp. UMB0728 (Accession No. NZ_PKLA01000016.1), *Brachybacterium* sp. UMB0905 (Accession No. NZ_PNHL01000013.1), *Microbacterium* sp. UMB0228 (Accession No. NZ_PNFU01000016.1), *Staphylococcus* sp. UMB0328 (Accession No. NZ_PNFS01000002.1), and *Streptococcus* sp. UMB0029 (Accession No. NZ_PNGD01000027.1).

As prior short-read 16S rRNA sequencing surveys have cataloged the taxa of the urobiome using either the V1-V3 regions, the V4 region, or the V4-V6 regions, we were interested in ascertaining the variable regions’ ability to correctly identify urinary microbes at the genus and species level. These regions were spliced from the 399 unique 16S rRNA near-full or full-length sequences and classified. Most classifications at the genus-level (often the depth to which short-read 16S rRNA gene sequence surveys report taxa) were correct; however, we identified a few sequences that were incorrectly or unable to be classified at the genus-level (**Table 2**). For all three variable regions considered (V1-V3, V4, and V4-V6), there were urinary species for which genus-level designations could not be made (**Table 2**, “Not classified”). Three sequences in our collection, *Peribacillus frigoritolerans* 462G, *Moraxella osloensis* UMB0416, and *Enterococcus raffinosus* UMB9185, were not correctly identified at the genus level by all three regions.

**Table 2:** Urinary species with a 16S rRNA gene sequence that was misclassified or not classified at the genus-level when considering a variable region(s).

Full-Length Sequence Classification	Variable Region Classification
V1-V3	
*Enterobacter bugandensis*	*Klebsiella michiganensis*
*Enterococcus raffinosus*	*Escherichia-Shigella coli*
*Escherichia coli*	Not classified
*Granulicatella adiacens*	Not classified
*Klebsiella aerogenes* (3)	*Raoultella planticola*
*Klebsiella oxytoca*	*Enterobacter cloacae*
*Limosilactobacillus reuteri*	HT002 nan
*Moraxella osloensis*	*Enhydrobacter* nan
*Peribacillus frigoritolerans*	*Bacillus simplex*
V4	
*Actinobaculum massiliense*	*Actinotignum schaalii*
*Dolosicoccus paucivorans*	Not classified
*Enterobacter bugandensis*	*Klebsiella pneumoniae*
*Enterobacter hormaechei* (2)	*Klebsiella pneumoniae*
*Enterococcus raffinosus*	*Escherichia-Shigella coli*
*Enterococcus faecalis*	*Corynebacterium mycetoides*
*Limosilactobacillus reuteri*	HT002 nan
*Micrococcus terreus*	Not classified
*Moraxella osloensis*	*Enhydrobacter* nan
*Peribacillus frigoritolerans*	*Bacillus* simplex
*Pseudoclavibacter alba*	Not classified
V4-V6	
*Citrobacter koseri*	Not classified
*Enterobacter hormaechei* (2)	*Klebsiella aerogenes*
*Enterococcus raffinosus*	*Escherichia-Shigella dysenteriae*
*Enterococcus faecalis*	*Corynebacterium* nan
*Moraxella osloensis*	*Enhydrobacter* nan
*Peribacillus frigoritolerans*	*Bacillus* simplex

For instances in which multiple sequences of the same species were misclassified, the number of misclassified sequences is indicated in parentheses after the species name. “Not classified” signifies that the genus could not be determined, and “nan” indicates that the species designation could not be made.

When species predictions were considered (i.e., genus prediction was correct), we found that 100 designations were incorrect for the V1-V3 region (25.06%), 142 for the V4 region (35.59%), and 112 for the V4-V6 region (28.07%) (**Figure 1**). Many of these sequences were unable to be resolved at the species level. In these cases, the genus prediction was correct, but the species was not predicted. This includes 53 sequences for the V1-V3 region, 47 for the V4 region, and 52 for the V4-V6 region. **Supplementary Table S3** lists each of the 399 sequences in the data set, including if their V1-V3, V4, and V4-V6 variable region classification resulted in a genus match (mismatched species), a genus and species match, or a genus and species mismatch. In the case of a mismatch (either genus or species), the predicted taxon is listed. **Table 3** summarizes these results for the UTI-associated bacterial species *E. coli*, *Klebsiella pneumoniae*, *Proteus mirabilis*, *Morganella morganii*, *Staphylococcus aureus*, and *Pseudomonas aeruginosa*.

**Table 3:** Accuracy of prediction of sequences of UTI-associated species at the genus-level and species-level for the V1-V3, V4, and V4-V6 regions.

Species	No. Sequences	V1-V3		V4		V4-V6	
		Genus	Species	Genus	Species	Genus	Species
*E. coli*	26	25	21	26	26	26	22
*E. faecalis*	4	4	4	3	0	3	3
*K. pneumoniae*	25	25	16	25	25	25	24
*M. morganii*	3	3	3	3	3	3	2
*P. aeruginosa*	2	2	2	2	2	2	2
*S. aureus*	4	4	3	4	4	4	3

## Discussion

The data set presented here provides a resource for researchers investigating the bacterial diversity of the urinary tract. As most studies of this diversity to date have employed short-read sequencing targeting 16S rRNA gene sequence variable regions, our analyses into the “correctness” of such classifications enables a new interpretation of prior studies of the female urinary microbiome that have targeted the V1-V3, V4, or V4-V6 variable regions. As variable region studies often report taxa at the genus-level, we can first consider those sequences that resulted in a misclassification (or the inability to be classified) at the genus-level. It is important to note that not all of the sequences for the species listed in **Table 2** were misclassified/not classified. Nonetheless, this table does include species associated with infection or symptoms or the lack thereof. As more urinary bacteria representatives are included in training models for taxonomic classification, one would anticipate that such instances of misclassification or failed classifications would be reduced. Exceptions, however, would be those species that have nearly identical 16S rRNA gene sequences, e.g., species of lactobacilli, species of *Gardnerella* (34–36).

16S rRNA gene sequences of the closely related *Enterobacter bugandensis* (n=1) and *Enterobacter hormaechei* (n=2) were misclassified as *Klebsiella pneumoniae* based on the V4 region (**Supplementary Table S3**). Strains of these closely related *Enterobacter* species often exhibit multidrug-resistance (MDR) (48, 49). Prior studies have identified MDR strains of *E. hormaechei* in urine samples from individuals with UTI symptoms (50–52). Strains of *K. pneumoniae*, a common cause of UTIs due to indwelling catheters, are also frequently MDR (see reviews (53, 54)). The *E. bugandensis* 16S rRNA gene sequence was also misclassified as *Klebsiella michiganensis* (V1-V3) and *Klebsiella aerogenes* (V4-V6); while both of these *Klebsiella* species similarly have been shown to have MDR, only *K. aerogens* has been associated with UTIs (55–57). Misclassification of these two *Enterobacter* species by variable region analysis may underestimate its contribution to UTIs and/or be misleading with regards to the antibiotic resistances present in a sample. While 16S rRNA gene surveys are not a clinical tool, they are frequently used when investigating associations between urobiome constituents and lower urinary tract symptoms (see review (6)).

*Enterococcus faecalis* and *Enterococcus faecium* also have been associated with catheter-associated UTIs (CAUTIs) (58). One of the four *E. faecalis* sequences was misclassified as *Corynebacterium* based on the V4 and V4-V6 regions. While the other three sequences in the data set were predicted as *Enterococcus* by their V4 region, they were assigned to the species *E. faecium* (**Figure 1**). Recent studies of urinary strains of *E. faecalis* and *E. faecium* have found that the species vary in their resistances to antibiotics (58–61). Thus, studies that target the V4 region are limited to a genus-level classification of *Enterococcus* at best.

As shown in **Table 3**, most of the other UTI-associated bacterial species are correctly identified at the genus level. Furthermore, false identifications of sequences of *M. morganii* and *P. aeruginosa* did not occur for the three regions examined here. Only one sequence – *Enterococcus raffinosus* UMB918 – was misidentified as *E. coli* based on the V1-V3 and V4 regions (**Supplementary Table S3**). In addition to the misidentified *E. hormaechi* and *E. bugandensis* sequences previously mentioned, four *K. aerogenes* and four *K. quasipneumoniae* sequences were predicted to be *K. pneumoniae* based on the V4 region; the V1-V3 and V4-V6 sequence species predictions for these other *Klebsiella* sequences were, however, correct. The V4 sequences of many of the *Staphylococcus* species, including *S. capitis*, *S. epidermidis*, *S. haemolyticus*, *S. hominis*, *S. lugdunesis*, *S. pasteuri*, and *S. warneri*, were incorrectly predicted to be *S. aureus* (**Supplementary Table S3**). When the expanded V4-V6 region was considered, only three of the four *S. haemolyticus* sequences were erroneously identified as *S. aureus*. Therefore, we can conclude that urobiome studies – particularly those considering the V4 region alone – may be overestimating the relative abundance of *K. pneumoniae* and *S. aureus*.

Examination of symptom-associated species alone limits our understanding of the urobiome. Numerous studies have associated *Lactobacillus* species with a lack of lower urinary tract symptoms in females (19, 22, 39, 62, 63). The V1-V3, V4, and V4-V6 regions were unable to correctly assign species designations for many of the *L. iners*, *L. crispatus*, and *L. gasseri* sequences (**Supplementary Table S3**). These three species are the most frequently detected lactobacilli by the EQUC method among continent females (39). Several *L. crispatus* strains have shown the ability to inhibit or kill uropathogenic *E. coli* strains (64–66). In contrast, *L. gasseri*, *L. iners*, and *L. jensenii* are frequently found in the urobiomes of females with urinary incontinence (19, 39). Thus, distinguishing between these *Lactobacillus* species is key to understanding continence in females. The V1-V3 regions performed best in distinguishing the urinary *Lactobacillus* species, correctly identifying *L. crispatus*, *L. iners*, *L. jensenii*, *L. mulieris*, and most *L. gasseri* (11/12); *L. paragasseri* strains were predicted to be *L. gasseri* (**Table 2**), which is not surprising given the significant sequence similarity of the 16S rRNA gene sequences for these sister taxa (34). In our collection, *L. paragasseri* and *L. gasseri* 16S rRNA gene sequences can differ by as few as 1 nucleotide. Thus, urobiome studies that target the V1-V3 region are best for reliably determining *Lactobacillus* species diversity.

While prior work assessing the capability of variable regions to resolve urinary species did find that NCBI’s database outperformed SILVA, which was used here, this prior assessment was conducted using a previous version of SILVA (v132) (33). A greater precision was observed here, for a larger and more diverse representation of 16S rRNA gene sequences from urinary isolates, than previously reported for this prior version of SILVA (33). Searching the SILVA database used here (v138.1) found that many of the taxa included in our reference data set had numerous representative sequences, highlighting that these databases are only as good as the data publicly available. We will note that after our analyses, we did reinvestigate the taxonomic designations (see Methods) as taxonomies are amended as new information emerges. We chose to use the SILVA database here as most prior 16S rRNA gene sequence surveys of the urobiome have relied on this database, albeit prior versions. It, along with the NCBI collection, are “preferred” databases per the Urobiome Consensus (32). Our data set will contribute to improving these databases. Likewise, additional closed genome sequences for members of the urobiome also will improve representation of genetic diversity of species within this niche.

As our analysis shows, it is possible to reliably characterize the urobiome at the genus level via short read sequencing. However, there are several cases in which distinguishing between two species of the same genera is critical for interpretation. Each of the regions considered here have their own limitations for distinguishing species. Concurring with prior evaluations of the female urobiome characterization by 16S rRNA gene sequence variable regions, we found the V1-V3 region to more accurately resolve taxa than V4-V6 region or V4 region alone (33). However, a similar study of bacteria of the male urobiome identified the V1-V2 amplicon as more precise (67). This highlights the importance of selecting a variable region(s) that can accurately capture the bacterial diversity present in the specific niche being explored. Informed decisions thus necessitate routine assessments of the strengths and limitations of individual variable regions as sequencing platforms, databases, and/or bioinformatic tools improve (37, 68, 69).

Nevertheless, short-read sequencing studies have inherent limitations, which can be overcome by using full-length 16S rRNA gene sequences. With decreasing error rates and costs associated with long-read sequencing technologies, full-length 16S rRNA gene sequencing can reliably resolve species and even strains (70). Full-length 16S rRNA gene sequencing surveys have been instrumental in identifying new 16S rRNA gene variants, e.g., those in the vaginal microbiome (71), bacterial transmission, e.g., mother-to-infant transmission of oral bacteria (72), and temporal dynamics, e.g., gut microbiota post-antibiotic treatment (73). Conducting such full-length 16S rRNA gene sequence surveys of the urobiome have only recently been explored (74). Additional studies are needed to improve the resolution of the bacterial constituents of the urobiome. The 96 sequences generated here have been deposited in GenBank. The data set presented here (**Supplementary Data Sheet S2**) can be used to supplement existing databases, increasing urobiome species variation for culturable constituents of the urobiome. Full-length 16S rRNA gene sequencing of the urobiome is needed for capturing those members of the urinary community that cannot be grown in the lab.

## Data availability statement

The data presented in the study are deposited in GenBank, accession numbers OR975923 through OR976020; accession numbers for all data included in the analyses presented here can be found in **Supplementary Table S1**.

## Author contributions

GB: Formal analysis, Writing – original draft, Writing – review & editing, Data curation, Investigation. AE: Data curation, Formal analysis, Investigation, Writing – review & editing, visualization. ZM: Data curation, Formal analysis, Investigation, Writing – review & editing. SB: Formal analysis, Writing – review & editing. AW: Writing – review & editing, Conceptualization. CP: Conceptualization, Writing – review & editing, Formal analysis, Writing – original draft.
